# Supplementary figures and images for: Identification of Zip8-correlated hub genes in pulmonary hypertension by informatic analysis
Source: PeerJ. 2023 Aug 28;11:e15939. doi: 10.7717/peerj.15939 (PMC10470448; doi:10.7717/peerj.15939)

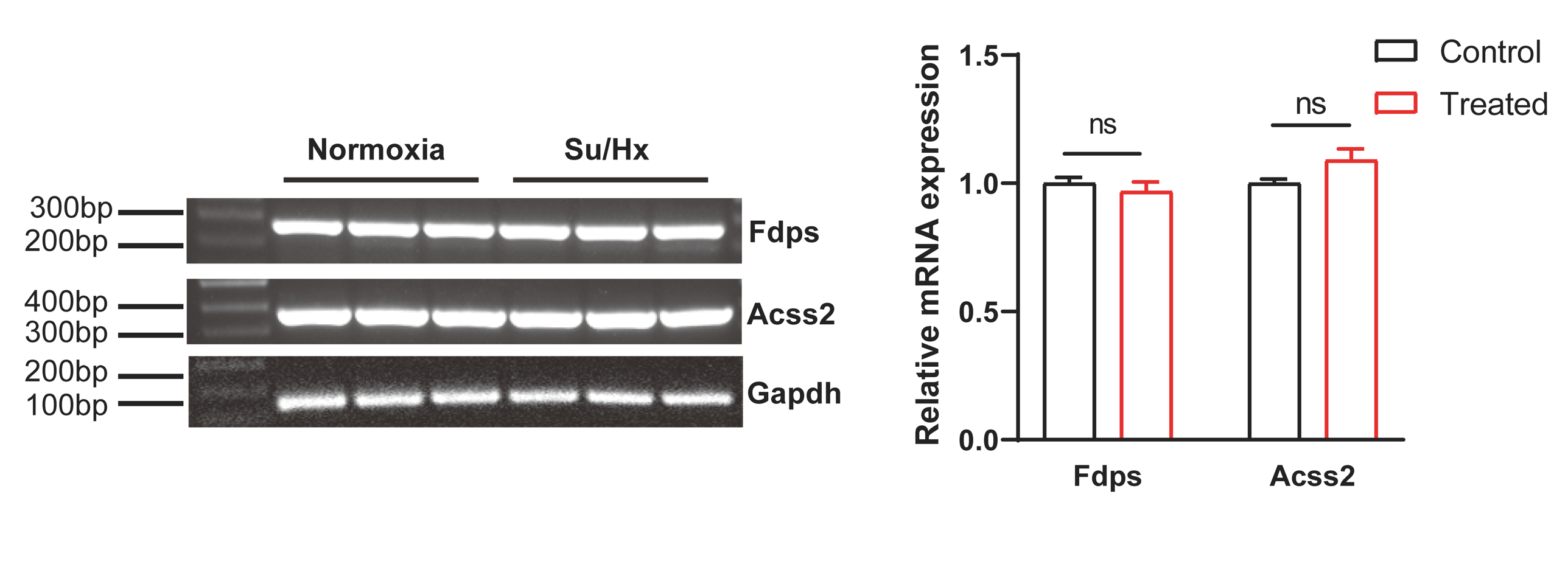

Supplement: Supplemental Information 1 — n = 10, Data are shown as mean ± SEM; ns, no significance. [file peerj-11-15939-s001.png]
