# Supplementary material for: Identification of Zip8-correlated hub genes in pulmonary hypertension by informatic analysis
Source: PeerJ. 2023 Aug 28;11:e15939. doi: 10.7717/peerj.15939 (PMC10470448; doi:10.7717/peerj.15939)
Supplement: Supplemental Information 2 [file peerj-11-15939-s002.docx]

ARRIVE 2.0 Checklist

1. Study Design

See the materials & and methods section in line 92-103 for details.

1. Sample size

See the materials & and methods section in line 95 and 100 for details.

1. Inclusion and exclusion criteria

See the materials & and methods section in line 101 and 102 for details.

1. Randomisation

See the materials & and methods section in line 94 for details.

1. Blinding/Masking

See the materials & and methods section in line 103 details.

1. Outcome measures

See the materials & and methods section in line 110-117 details

1. Statistical methods

See the materials & and methods section in line 137-139 details

1. Experimental animals

See the materials & and methods section in line 93-94 for details

1. Experimental procedures

See the materials & and methods section in line 94-106 for details

1. Results

See the materials & and methods section in line 219-237 for details

1. Abstract

See the abstract section in line 28-48 for details

1. Background

See the abstract section in line 28-33 for details

1. Objectives

See the introduction section in line 73-74 for details

1. Ethical statement

See the materials & and methods section in line 107-109 for details

1. Housing and husbandry

See the materials & and methods section in line 94-101 for details

1. Animal care and monitoring

See the materials & and methods section in line 95-103 for details

1. Interpretation/scientific implications

See the discussion section in line 281-289 for details

1. Generalisability/translation

See the conclusion section in line 310-316 for details

1. Protocol registration

See the materials & and methods section in line 92-109 for details

1. Data access

See the availability of data section in line 320-322 for details

1. Declaration of interests

See the declaration of interests section in line 323-324 for details
